# THE NEW MAP OF LIFE: TRANSFORMING THE COURSE OF LONG LIVES THROUGH IMPACT-DRIVEN RESEARCH

**DOI:** 10.1093/geroni/igae098.0558

**Published:** 2024-12-31

**Authors:** Yochai Shavit, Laura Carstensen

**Affiliations:** Chair; Discussant

## Abstract

The global rise in life expectancy and the emergence of societies that are more age-diverse than ever before in human history call for a re-design of the life course.The Stanford Center on Longevity released the New Map of Life- a report detailing principles to guide research, policies, and interventions aimed at improving long lives and age-diverse societies, taking advantage of the opportunities these demographic changes entail. These have already influenced recommendations by key organizations, including the National Academy of Medicine and the World Economic Forum. This symposium offers the first look into research findings guided by the New Map of Life and their translation into actionable recommendations. First, Weiss presents an innovative approach to model long life trajectories based on Artificial Intelligence that outperforms current life expectancy and morbidity calculators. On the population level, Ashwin presents macroeconomic challenges and advantages of longevity, highlighting differences between aging societies and long-living societies. Growney describes a pilot program leveraging older adults’ strengths in early childhood education classrooms. Rangan presents findings from a study of graduating medical students highlighting the need to revamp medical education to address a severe lack of training in older adult medical care. Finally, Chu presents focus-group findings exploring older adults’ technological needs and challenges in daily life, which can alter aging experiences. All presenters detail specific ways in which their findings can inform stakeholders’ actions. Prof. Carstensen will conclude by discussing the broad picture emerging from these studies and their importance for further development of the framework.

